# Conditional gene deletion with DiCre demonstrates an essential role for CRK3 in *L* *eishmania mexicana* cell cycle regulation

**DOI:** 10.1111/mmi.13375

**Published:** 2016-04-13

**Authors:** Samuel M. Duncan, Elmarie Myburgh, Cintia Philipon, Elaine Brown, Markus Meissner, James Brewer, Jeremy C. Mottram

**Affiliations:** ^1^ Wellcome Trust Centre for Molecular Parasitology, Institute of Infection, Immunity and Inflammation, College of Medical, Veterinary and Life SciencesUniversity of GlasgowGlasgowG12 8TAUK; ^2^ Centre for Immunology and Infection, Department of BiologyUniversity of York, Wentworth WayHeslingtonYorkYO10 5DDUK

## Abstract

*Leishmania mexicana* has a large family of cyclin‐dependent kinases (CDKs) that reflect the complex interplay between cell cycle and life cycle progression. Evidence from previous studies indicated that Cdc2‐related kinase 3 (CRK3) in complex with the cyclin CYC6 is a functional homologue of the major cell cycle regulator CDK1, yet definitive genetic evidence for an essential role in parasite proliferation is lacking. To address this, we have implemented an inducible gene deletion system based on a dimerised Cre recombinase (diCre) to target CRK3 and elucidate its role in the cell cycle of *L. mexicana*. Induction of diCre activity in promastigotes with rapamycin resulted in efficient deletion of floxed *CRK3*, resulting in G2/M growth arrest. Co‐expression of a *CRK3* transgene during rapamycin‐induced deletion of *CRK3* resulted in complementation of growth, whereas expression of an active site *CRK3* ^T178E^ mutant did not, showing that protein kinase activity is crucial for CRK3 function. Inducible deletion of *CRK3* in stationary phase promastigotes resulted in attenuated growth in mice, thereby confirming CRK3 as a useful therapeutic target and diCre as a valuable new tool for analyzing essential genes in *Leishmania*.

## Introduction

The leishmaniases, diseases caused by protozoan parasites of the genus *Leishmania*, have diverse clinical manifestations dependent on the species and host immune response. Leishmaniasis is a substantial public health issue, causing an estimated 40,000 deaths annually and approximately 0.2–0.4 and 0.7–1.2 million visceral and cutaneous manifestations of the disease respectively (Alvar *et al*., 2012). Existing drug therapies are problematic because of high treatment costs, toxicity and undesirable administration routes, making the development of novel and effective drug therapies to expand the current repertoire crucial. Phenotypic strategies to identify drug targets in the mammalian infective amastigote life cycle stage are of particular importance for drug discovery programs.

As unicellular organisms, *Leishmania* depend on stringent control of cellular division to propagate and maintain infection. Protein kinases elicit pronounced effects on the *Leishmania* cell cycle by regulation of cell signalling pathways, and a number of protein kinases have been identified that are essential for promastigote viability (Dacher *et al*., 2014; Wang *et al*., 2005). The cyclin‐dependent kinases (CDK) are of particularly interest because of their pivotal roles as cell cycle regulators. The use of CDK inhibitors in cancer therapy (Cicenas and Valius, 2011; Knapp and Sundström, 2014) and the relative expansion of this protein family in *Leishmania* relative to other unicellular organisms distinguishes them as suitable drug targets. In particular, the CDK‐related kinase CRK3 has been demonstrated as being important for regulation of the *L. mexicana* promastigote cell cycle by existing genetic manipulation techniques and cell cycle arrest following treatment with CDK inhibitors (Hassan *et al*., 2001; Grant *et al*., 1998; 2004). Recombinant protein kinase activity assays (Gomes *et al*., 2010) and yeast recovery mutants (Wang *et al*., 1998) have provided further validation of CRK3 as a drug target, leading to the identification and synthesis of a number of CRK3 inhibitors (Cleghorn *et al*., 2011; Goyal *et al*., 2014; Grant *et al*., 2004; Řezníčková *et al*., 2015; Walker *et al*., 2011). Regulation of CRK3 expression in *L. mexicana* is desirable to further assess its function in both procyclic promastigote and amastigote life cycle stages, however, no system exists for conditional deletion of essential genes. Recent application of plasmid shuffle methodology has addressed this issue by enabling the generation of partial *null* mutants to further study essentiality and important residues within coding sequences (Dacher *et al*., 2014; Morales *et al*., 2010), however, the gene is not deleted and this prevents phenotyping of a *null* mutant.

To address this limitation, we have implemented a rapamycin‐inducible gene deletion system using a dimerised Cre recombinase (diCre) (Andenmatten *et al*., 2013; Collins *et al*., 2013; Jullien *et al*., 2003) to target *CRK3* and elucidate its role in the cell cycle of *L. mexicana*. *L. mexicana* is generally diploid (Rogers *et al*., 2011) and both *CRK3* alleles were replaced with a ‘floxed’ *CRK3* open reading frame and the diCre coding sequence through promastigote transfection and homologous recombination. This system was used to conditionally delete *CRK3* during promastigote growth and so prove that CRK3 mediates the transition through G2/M. Induced loss of *CRK3* was complemented by expression of a *CRK3* transgene but not by expression of an inactive site (T178E) *CRK3* mutant, showing that protein kinase activity is crucial for CRK3 function. Significantly, conditional deletion of *CRK3* in stationary phase promastigotes and subsequent attenuation during murine infection demonstrates that CRK3 activity is essential for establishing infection. This system represents a new method to directly assess whether a gene is essential to parasite viability and provides novel insight into the function of essential genes in *Leishmania*.

## Results

### DiCre activity is tightly regulated in *L. mexicana* promastigotes and amastigotes

**Figure 1:**
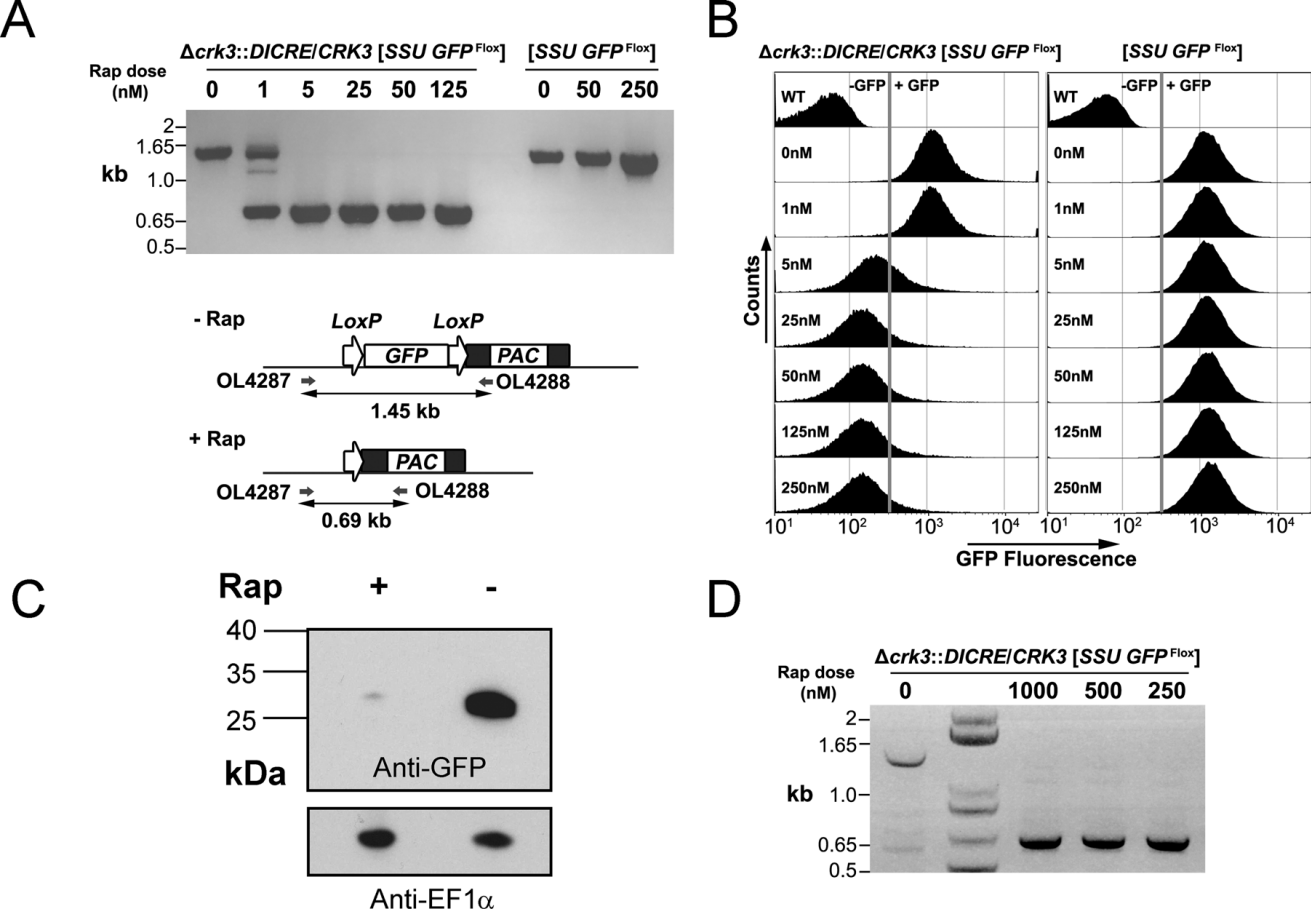
Validation of inducible diCre in *L. mexicana*: conditional deletion of *GFP* in promastigotes and amastigotes. A. Gene excision analyzed by PCR amplification. Schematic (lower) shows the *SSU GFP* ^Flox^ locus and the recombination event expected after treatment with rapamycin (Rap). (upper) PCR amplification with oligonucleotides 4287 and 4288 from experimental (Δ*crk3*::*DICRE*/*CRK3* [*SSU GFP* ^Flox^]) and control [*SSU GFP* ^Flox^] promastigotes at 5 days post‐treatment with different concentrations of rapamycin. B. Flow cytometry assessment of GFP intensity of experimental and control promastigotes incubated in the presence or absence of rapamycin for 5 days. C. Western blotting analysis with anti‐GFP and anti‐EF1α loading control antibodies of protein extracted from experimental promastigotes grown for 5 days in the presence or absence of 100 nM rapamycin. D. PCR analysis of *GFP* ^Flox^ loss (as described in A) in amastigotes after 24 h rapamycin treatment (0–1000 nM), followed by 120 h infection in bone‐marrow derived macrophages. Lane 2 contains a 1 kb+ DNA ladder.

To test the activity of diCre in *L. mexicana* promastigotes, a reporter cell line was generated by integration of a loxP‐flanked *GFP* into the ribosomal locus: [*SSU GFP* ^Flox^]. This cell line was transfected with a diCre construct containing the two dimerizable Cre recombinase subunits with the homologous flanks of *crk3* to generate the heterozygous line (Δ*crk3*::*DICRE*/*CRK3* [*SSU GFP* ^Flox^]). Integration of the diCre construct at the *CRK3* locus was confirmed by PCR analysis (Fig. S1A). No effect on the growth of *SSU GFP* ^Flox^ or Δ*crk3*::*DICRE*/*CRK3* [*SSU GFP* ^Flox^] was observed in the presence of the dimerization ligand, rapamycin, up to the highest dose of 250 nM (Fig. S1B). *GFP* excision following incubation with increasing concentrations of rapamycin was investigated by PCR using specific primers flanking GFP. A single 1.45 kb PCR product, the floxed GFP fragment, was detected in the absence of rapamycin, whilst a 0.69 kb PCR product, representing the excised locus, was detected following rapamycin treatment only (Fig. 1A), indicating tight regulation of diCre activity. Δ*crk3*::*DICRE*/*CRK3* [*SSU GFP* ^Flox^] and [*SSU GFP* ^Flox^] promastigotes grown for 5 days in the presence or absence of increasing concentrations of rapamycin were analyzed by flow cytometry to measure levels of GFP expression (Fig. 1B). Treatment of Δ*crk3*::*DICRE*/*CRK3* [*SSU GFP* ^Flox^] promastigotes with greater than 5 nM rapamycin resulted in substantial loss of GFP expression compared with the untreated controls, whilst GFP expression in [*SSU GFP* ^Flox^] was the same following growth in all concentrations of rapamycin. GFP loss in Δ*crk3*::*DICRE*/*CRK3* [*SSU GFP* ^Flox^] promastigotes grown in the presence or absence of 100 nM rapamycin for 5 days was further assessed by Western blotting of total protein extracts using anti‐GFP antibody (Fig. 1C). Rapamycin‐treated promastigotes had considerably reduced GFP compared with the untreated controls, thereby demonstrating that gene loss results in reduced protein expression. These data also demonstrate that expression of *diCre* from the *CRK3* locus is sufficient to efficiently excise the *GFP* transgene at rapamycin concentrations above 5 nM, and that no background diCre activity can be detected in the absence of ligand. About 100 nM of rapamycin was chosen as the optimum concentration to induce diCre activity in promastigotes whilst having no effect on *in vitro* cell growth.

To test diCre functionality in amastigotes, infectious promastigotes of the experimental line Δ*crk3*::*DICRE*/*CRK3* [*SSU GFP* ^Flox^] were inoculated into BALB/c footpads and amastigotes purified from the resulting lesion. *Ex vivo* amastigotes retained high levels of green fluorescence and were incubated with rapamycin for 24 h in Schneider's medium prior to infection of bone‐marrow derived macrophages. Efficient excision of *GFP* ^Flox^ was detected by PCR amplification of a 0.69 kb fragment representative of *GFP* loss in all rapamycin treated samples (Fig. 1D) and GFP^‐^ (non‐fluorescent) amastigotes were observed by comparing images obtained through fluorescence live cell imaging (Fig. S1C). Residual GFP^+^ amastigotes were still visible by microscopy (Fig. S1C) and could be detected by flow cytometry (Fig. S1D); this was possibly because of the slow replication rate of amastigotes leading to a low rate of GFP turnover. These data demonstrate inducible diCre activity in amastigotes.

### Inducible deletion of CRK3 in *L. mexicana* promastigotes

**Figure 2:**
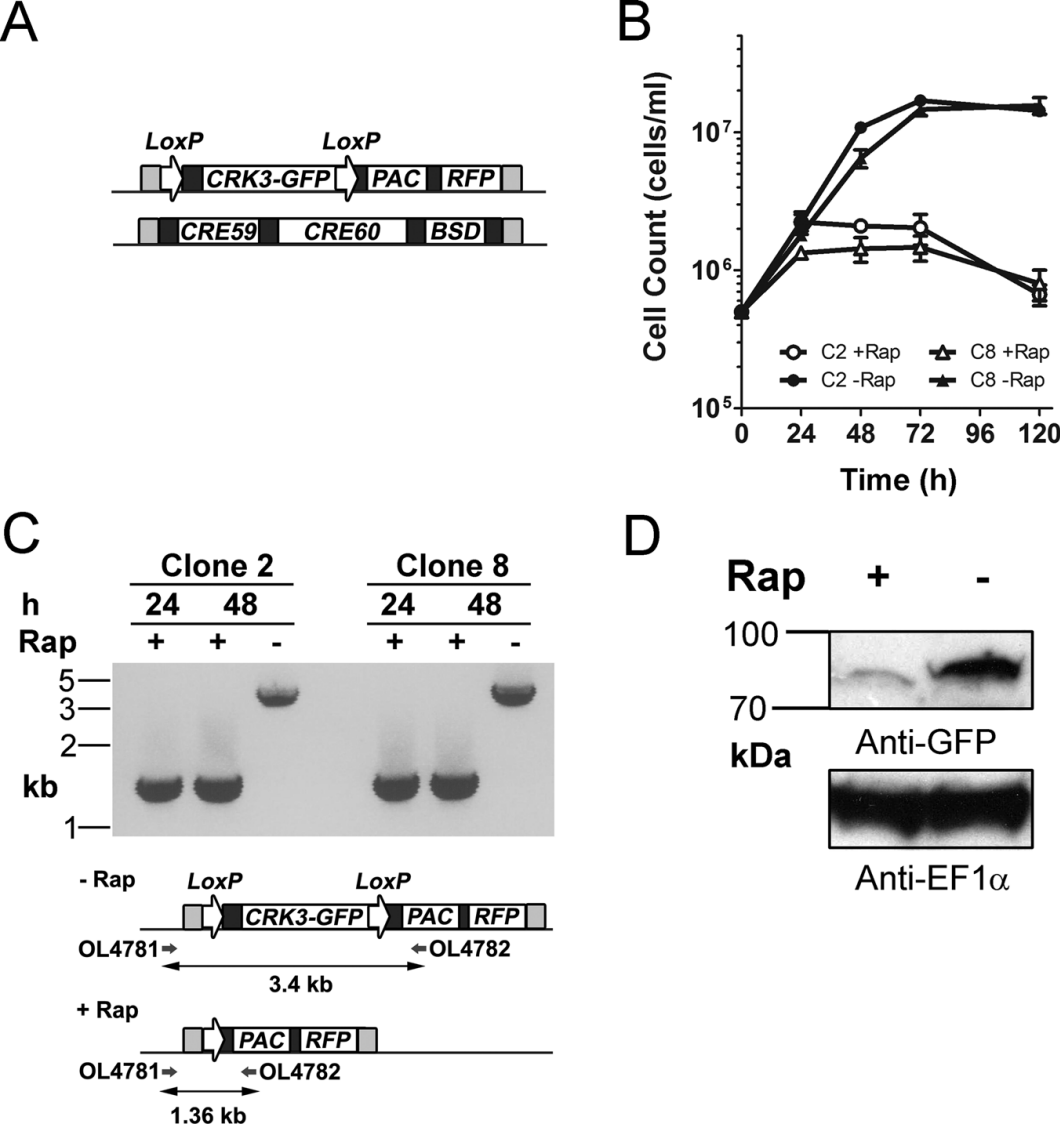
Generation of a *CRK3* conditional deletion cell line. A. Schematic showing the replacement of endogenous *CRK3* to generate Δ*crk3*::*DICRE*/Δ*crk3*::*CRK3* ^Flox^. One allele contains a *loxP* flanked *CRK3‐GFP* coding sequence with mCherry red‐fluorescent protein cassette (*RFP*) and puromycin drug selectable marker (*PAC*). The other allele contains genes encoding both diCre subunits (*CRE59, CRE60*) each linked with rapamycin binding domains (not shown: *FKBP12 and FRB* respectively) and a blasticidin resistance cassette (*BSD*). Each construct was flanked with 500 bp arms of homology (light grey) by Gateway recombination to facilitate integration at the *CRK3* locus. All coding sequences are flanked by regulatory elements (dark grey). *L. mexicana* parasites were transfected sequentially with the *diCre* construct and floxed *CRK3* to confer resistance to blasticidin and puromycin antibiotics respectively. B. Clones 2 and 8 promastigotes were seeded at a density of 5 × 10^5^ cells mL^−1^ and grown in the presence or absence (+/−) of 100 nM rapamycin for 5 days. Cell density was determined by counting at 24 h intervals and mean ± SD of triplicate values was plotted. C. (lower) A schematic representation of the floxed *CRK3* locus after excision. PCR amplification shows the primers binding upstream of the 5′ *CRK3* homologous flank and within the *PAC* cassette. (upper) PCR amplification of clones 2 and 8 at 24 h and 48 h +/−100 nM rapamycin treatment was conducted and the resulting amplicons resolved on an agarose gel. D. Western blotting analysis with anti‐GFP and anti‐EF1α loading control antibodies of protein extracted from experimental clone 2 promastigotes grown for 4 days in the presence or absence of 100 nM rapamycin.

The functional and efficient levels of diCre‐mediated excision of *GFP* underpinned the development of a system for conditional deletion of essential genes. Gateway recombineering was used to flank appropriate diCre and loxP expression constructs with gene‐specific, homologous flanks (Fig. S2). Plasmids were generated by this method to replace the two alleles of *CRK3*, an essential gene in *L. mexicana* (Hassan *et al*., 2001) (Fig S3A). The first allele of *CRK3* was replaced with *DICRE* (Δ*crk3*::*DICRE*/*CRK3*) and the second allele of *CRK3* was subsequently replaced with a floxed C‐terminal GFP‐tagged *CRK3* version (Δ*crk3*::*DICRE*/Δ*crk3*::*CRK3* ^Flox^; Figs. 2A and S3B). In addition, an *mCherry* red fluorescent protein coding sequence was incorporated downstream from the floxed *CRK3‐GFP* to facilitate flow cytometry and microscopy analysis. Transfection resulted in multiple clones with the expected genetic modifications, as confirmed by PCR analysis (Fig. S3B).

The growth of promastigotes from two Δ*crk3*::*DICRE*/Δ*crk3*::*CRK3* ^Flox^ clones were assessed following diCre‐mediated excision induced with 100 nM rapamycin (Fig. 2B). Cells were counted over the course of 5 days, revealing a pronounced growth defect and reduction in cell number in rapamycin‐treated cells compared with uninduced controls. PCR analysis of promastigotes grown in the presence or absence of 100 nM rapamycin for 24 and 48 h confirmed efficient loss of the *CRK3* gene (Fig. 2C) by the amplification of a single 1.36 kb DNA fragment for both rapamycin‐treated clones. The retention of the 3.4 kb amplicon containing the *CRK3* gene in both untreated clones is evidence that no background diCre activity can be detected in the absence of rapamycin. To test for loss of the CRK3‐GFP protein, total protein extracts of clone 2 promastigotes grown for 96 h in the presence or absence of 100 nM rapamycin were analysed by Western blot analysis with anti‐GFP antibody (Fig. 2D) Very low levels of protein were detected in the treated promastigotes compared to the untreated cells, confirming that the conditional gene loss leads to reduced protein levels. Treatment with 100 nM rapamycin did not result in any noticeable effect on *L. mexicana* promastigote growth (Fig. S1B), however the pronounced growth arrest arising from loss of the essential gene could possibly result in cellular stress that synergizes with rapamycin. These data show that this is a viable genetic manipulation strategy and that loss of CRK3 resulted in growth arrest and reduced cell numbers, both phenotypes consistent with loss of an essential gene.

### Cell cycle analysis of CRK3‐deficient promastigotes

**Figure 3:**
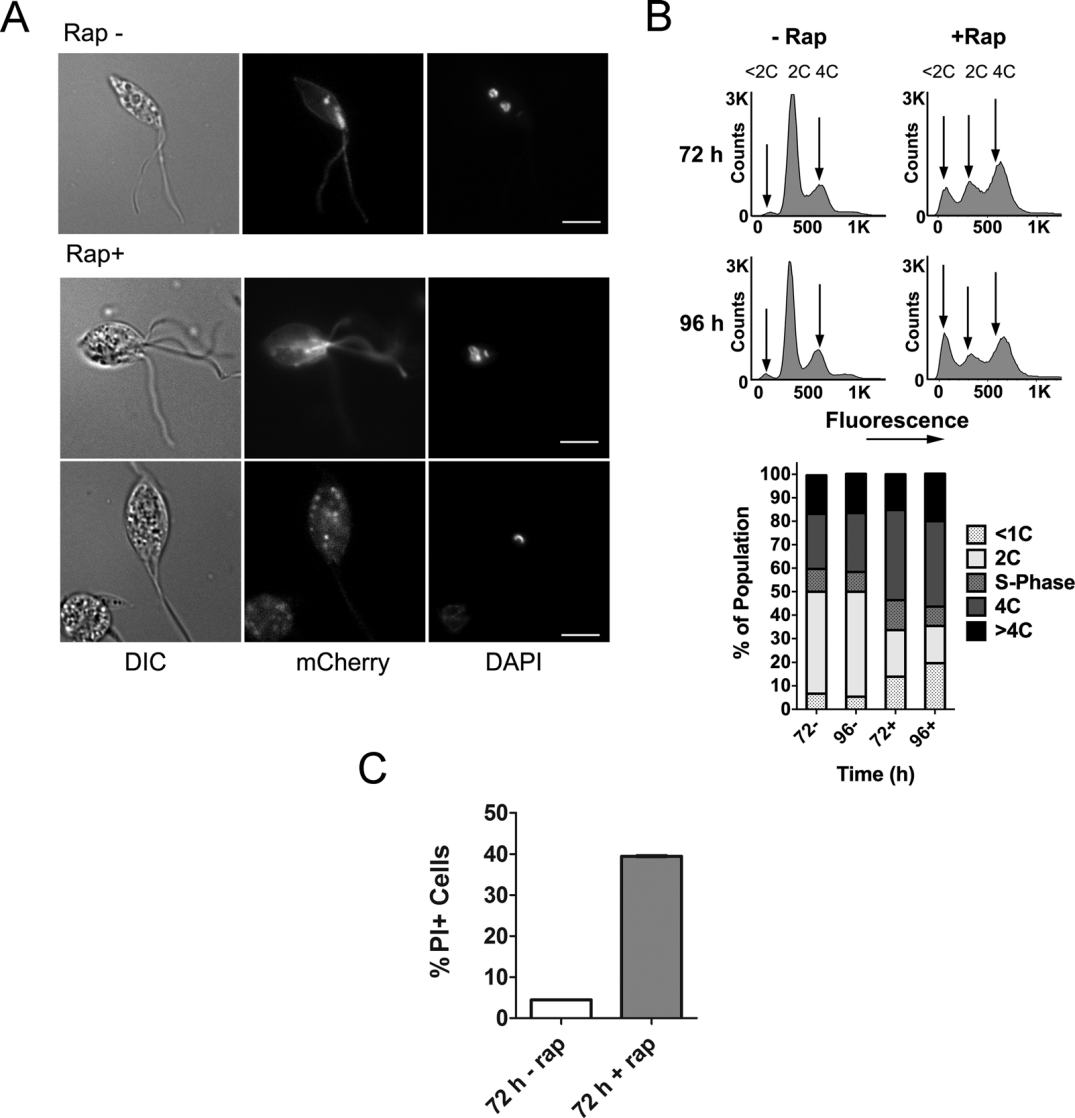
Analysis of *CRK3* deficient promastigotes. A. Representative images of cells grown in the absence (top) or presence (bottom two rows) of 100 nM rapamycin for 96 h. Promastigotes (clone 2) were stained with DAPI to observe nuclear and kinetoplast content alongside mCherry expression by fluorescence microscopy. Scale bar represents 5 μm. B. (upper) DNA content analysis of clone 2 promastigotes at 72 and 96 h post‐treatment. Cells were fixed with methanol and stained with propidium iodide for flow cytometry analysis of 100,000 cells to examine nuclear content. Arrows indicate the positions of cells in G1 phase (2C), in G2/M (4C) and low DNA content associated with increased incidence of <1C zoids. (lower) Graphical representation of the DNA content of each population based on the flow cytometry plots. C. The viability of cells grown in the absence (−) or presence (+) of 100 nM rapamycin for 72 h. Promastigotes (clone 2) were incubated with 5 µg mL^−1^ propidium iodide (PI) for 15 min and analyzed by flow cytometry. A heat lysed (HL) control in which half the sample was lysed by incubation at 70°C for 3 min was included to enable an appropriate live/dead gate to be drawn. Numbers represent the percentage of cells assessed as PI positive (PI+) based on the HL control. Data shown are the means of three technical replicates, data are representative of two independent experiments.

Previous attempts to impair CRK3 function in *Leishmania* by treatment with protein kinase inhibitors may have resulted in off‐target effects (Cleghorn *et al*., 2011; Efstathiou *et al*., 2014; Grant *et al*., 2004; Jorda *et al*., 2011; Reichwald *et al*., 2008; Řezníčková *et al*., 2015). Here the utilization of diCre mediated gene deletion enabled the effect of CRK3 depletion on the cell cycle to be investigated. Firstly, microscopic analysis of the cells at 96 h post‐induction showed an accumulation of large, aberrant cells with altered organelle homeostasis as evidenced by the presence of cells with multiple flagella (Fig. 3A). DAPI labelling of such multi‐flagellated cells to visualize cellular DNA revealed the presence of enlarged nuclei indicative of an arrest in mitosis. Interestingly, cells were also observed that lacked a nucleus but retained the kinetoplast (‘zoids’), a cell cycle defect observed previously by the treatment of promastigotes with CDK inhibitors (Grant *et al*., 2004). Secondly, flow cytometry was performed to determine the overall DNA content of Δ*crk3*::*DICRE*/Δ*crk3*::*CRK3* ^Flox^ promastigotes grown in the presence or absence of 100 nM rapamycin for 72 and 96 h (Fig. 3B). This analysis showed that conditional deletion of *CRK3* resulted in the accumulation of cells with 4C DNA content, associated with cell cycle arrest at G2/M, whilst an increasing population of cells with DNA content <1C indicates the accumulation of zoids. Finally, to assess the rate of cell death occurring in *CRK3*‐deficient cells a viability assay was performed on promastigotes after growth in the presence or absence of 100 nM rapamycin for 72 h (Figs. 3C and S4). After 72 h the proportion of propidium iodide positive cells (PI^+^) was around 40% indicating a high level of cell death, which likely resulted from the accumulation of anucleated zoids at this time point. Flow cytometry analysis of cell size (using forward scatter) was in agreement with the microscopy analysis and showed that *CRK3* deficient cells were substantially larger than cells retaining the gene (Fig. S4). Taken together, these data provide evidence that *CRK3* plays an essential role in regulating mitosis in replicating promastigotes.

### Active CRK3 is required for cell cycle progression in promastigotes

**Figure 4:**
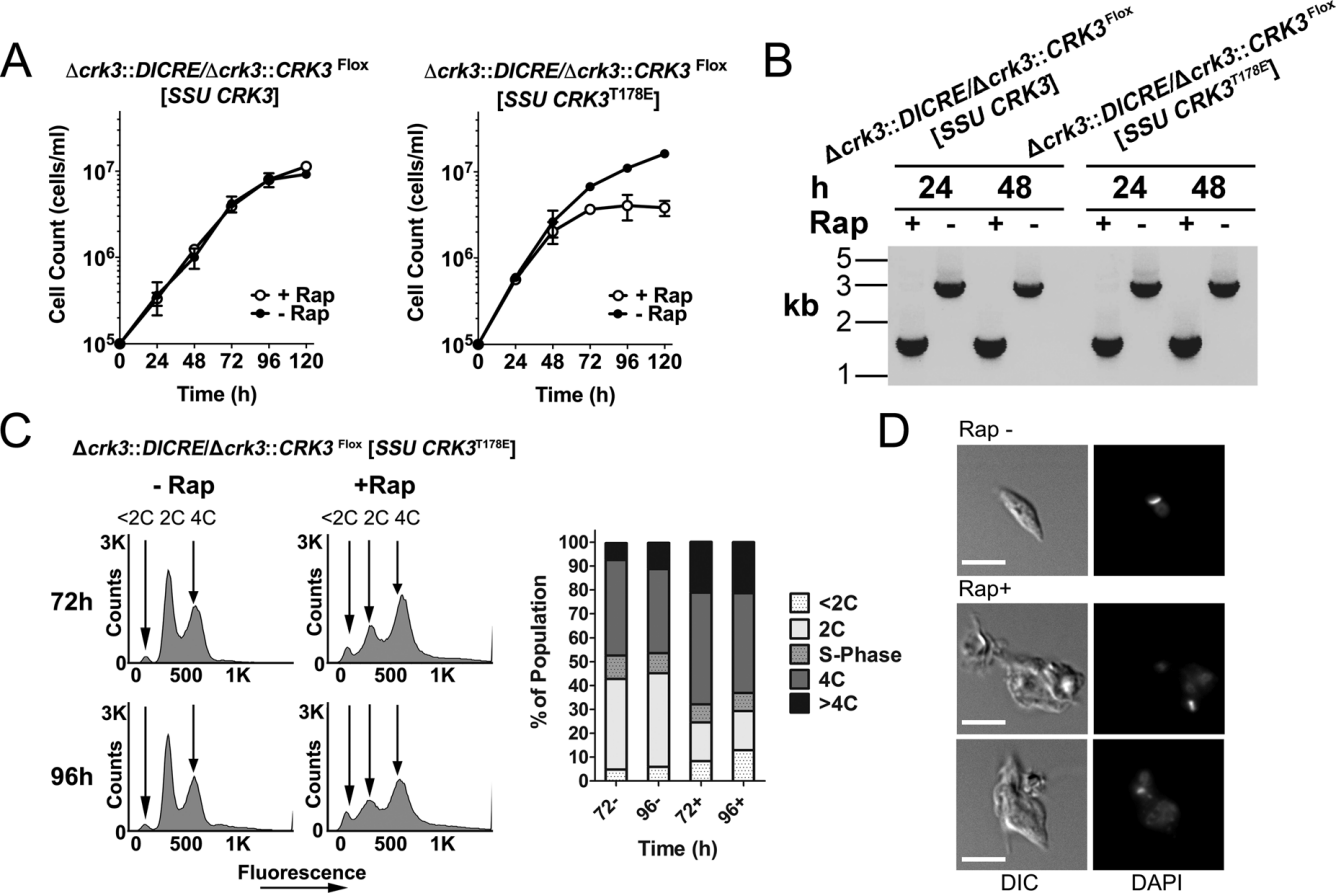
*CRK3* wild type and active site mutant complementation assays. A. Wild type complemented (Δ*crk3*::*DICRE*/Δ*crk3*::*CRK3* ^Flox^ [*SSU CRK3*], left graph) and mutant complemented (Δ*crk3*::*DICRE*/Δ*crk3*::*CRK3* ^Flox^ [*SSU CRK3* ^T178E^], right graph) cell lines were seeded as promastigotes at 1 × 10^5^ cells mL^−1^ and grown +/− 100 nM rapamycin for 5 days. Cell density was determined by counting at 24 h intervals and the mean ± SD of triplicate values was plotted. B. The resulting amplicons generated by PCR amplification of each cell line at 24 and 48 h after growth +/− 100 nM rapamycin. C. (left) DNA content analysis of Δ*crk3*::*DICRE*/Δ*crk3*::*CRK3* ^Flox^ [*SSU CRK3* ^T178E^] promastigotes after methanol fixation and staining with propidium iodide for flow cytometry analysis (100,000 cells) to examine nuclear content. Arrows indicate the positions of cells in G1 phase (2C), in G2 (4C) and low DNA content associated with increased incidence of <2C zoids. (right) Graphical representation of the DNA content of each population based on the flow cytometry analysis. D. Representative images of Δ*crk3*::*DICRE*/Δ*crk3*::*CRK3* ^Flox^ [*SSU CRK3* ^T178E^] promastigotes grown in the absence (top) or presence (bottom two rows) of 100 nM rapamycin for 96 h. Parasites were stained with DAPI to detect nuclear and kinetoplast DNA by fluorescence microscopy. Scale bar represents 5 μm.

We demonstrated that diCre could be used to efficiently delete a floxed copy of *CRK3*, so we exploited the efficiency of this system to further study gene function through complementation. Such a system was established by expressing a histidine‐tagged *CRK3* (*CRK3his*) (Hassan *et al*., 2001) transgene in Δ*crk3*:*DICRE*/Δ*crk3*::*CRK3* ^Flox^ promastigotes. No significant difference in growth was noted in the presence or absence of rapamycin over a 5 day period (Fig. 4A). Efficient excision of floxed *CRK3* in the induced culture was confirmed by PCR amplification of the diagnostic 1.36 kb fragment by 24 h post‐treatment with 100 nM rapamycin (Fig. 4B). The proliferation of promastigotes, despite loss of floxed *CRK3*, indicates *CRK3* transgene complementation in the induced Δ*crk3* cell line. Previous studies have shown that recombinant *L. mexicana* CRK3^T178E^ protein lacks H1 kinase activity (Gomes *et al*., 2010) and an *L. major CRK3* ^T178E^ mutant fails to complement a cdc2‐33(ts) yeast mutant (Wang *et al*., 1998). To test whether active CRK3 is required for cell growth, we exploited this complementation approach by generation of the cell line Δ*crk3*::*DICRE*/Δ*crk3*::*CRK3* ^Flox^ [*SSU CRK3* ^T178E^] expressing a T‐loop residue mutated version of *CRK3* from the ribosomal locus. Growth curves indicate that expression of the *CRK3* ^T178E^ transgene failed to complement the loss of *CRK3* ^Flox^ following induction with rapamycin (Fig. 4A and B) thereby demonstrating that CRK3^T178E^ cannot rescue loss of active CRK3. The overall growth rate of both complementation mutants was reduced relative to the parental line (Table 1) and may explain the growth arrest at 72 h following excision of *CRK3* in Δ*crk3*::*DICRE*/Δ*crk3*::*CRK3* ^Flox^ [*SSU CRK3* ^T178E^] compared with a more rapid onset of growth arrest in the parental line (Fig. 2B). These data show that active CRK3 is required for parasite growth. The *CRK3* deficient cells were analyzed by flow cytometry and fluorescence microscopy showing that Δ*crk3*::*DICRE*/Δ*crk3*::*CRK3* ^Flox^ [*SSU CRK3* ^T178E^] cells were blocked in G2/M (Fig. 4C) and were multi‐nucleate and aberrant (Fig. 4D). These data are in agreement with the phenotype observed following excision of *CRK3* in wild‐type cells (Fig. 3A and B), thereby indicating the importance of the T‐loop in regulating CRK3 activity. Based on these results, we conclude that transgene complementation can be used to confirm the specificity of conditional deletion of essential genes and also to probe the function of genes following mutagenesis.

**Table 1:** Comparisons of the growth rates of conditional *CRK3* deletion lines measured during logarithmic growth.

Experimental Line	Doubling Time (hrs)
Δ*crk3*::DICRE/Δ*crk3*::*CRK3* ^Flox^	10.6
Δ*crk3*::DICRE/Δ*crk3*::*CRK3* ^Flox^ [*SSU CRK3*]	15.5
Δ*crk3*::DICRE/Δ*crk3*::*CRK3* ^Flox^ [*SSU CRK3* ^T78E^]	13.6

### CRK3 is essential for *in vivo* infection of murine hosts

**Figure 5:**
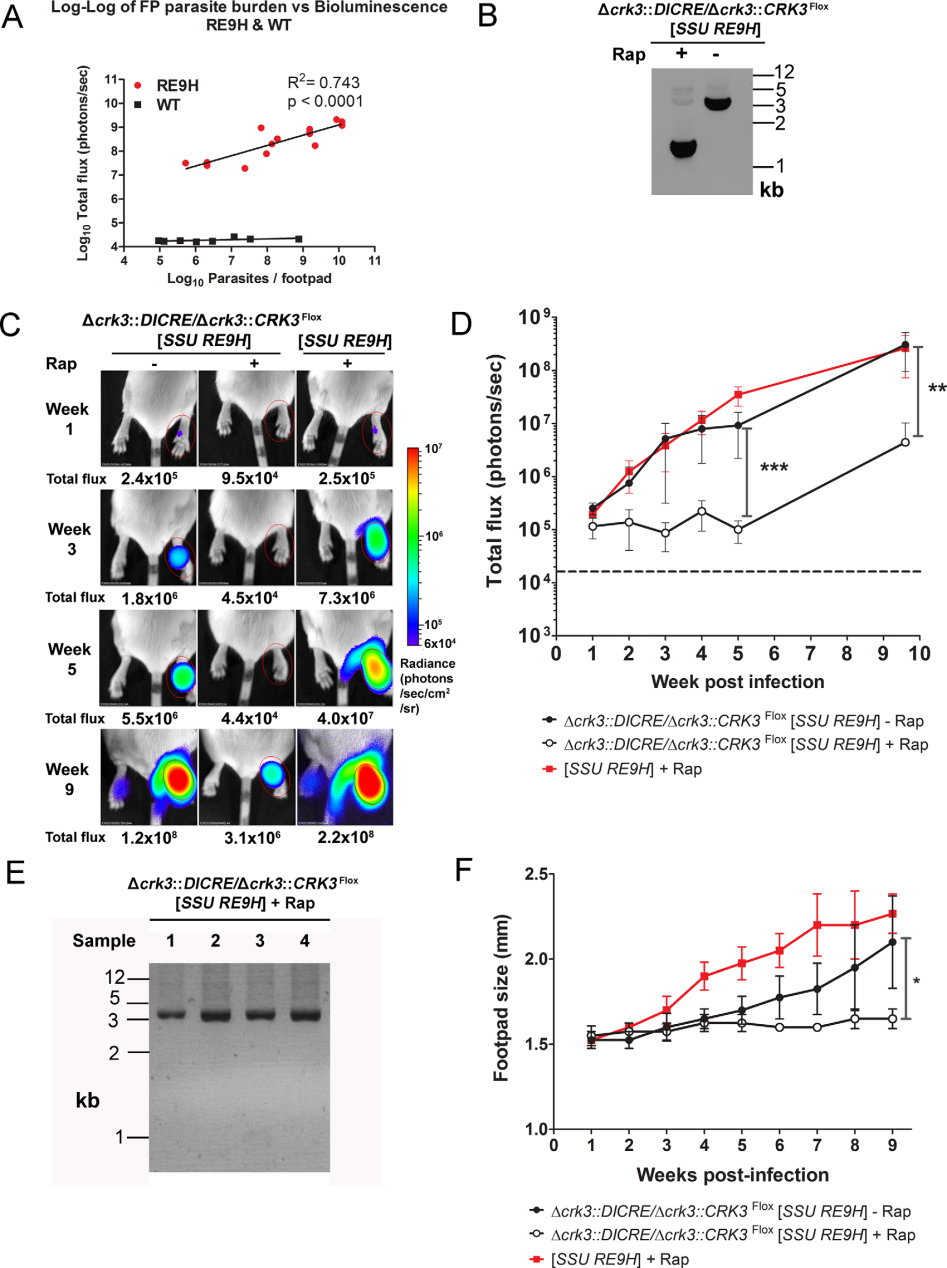
*CRK3* conditional deletion in stationary phase promastigotes and *in vivo* infection. A. Correlation between *in vivo* bioluminescence (total flux in photons per second) and parasite burdens from the same infected footpads. BALB/c mice were infected with *L. mexicana* WT or Ppy RE9H‐expressing stationary phase promastigotes and imaged weekly using an *in vivo* imaging system (IVIS). At 2, 4, 6 and 8 weeks post‐infection mice were sacrificed after imaging and parasite burdens in infected footpads determined using limiting dilution assays. Each point shows the total flux and parasite burden from the footpad in one mouse (*n* = 3–4 mice per time point). Linear regression line and *R^2^* was calculated from the log transformed data. B. PCR amplification of the floxed *CRK3* locus of Δ*crk3*::*DICRE*/Δ*crk3*::*CRK3* ^Flox^ [*SSU RE9H*] stationary phase promastigotes after incubation in the presence (+) or absence (−) of 1 μM rapamycin for 24 h. C. Control (−) or 24 h rapamycin‐treated (+) stationary phase promastigotes were inoculated into the footpads of BALB/c mice. The total flux (photons/s) emitted from the infected footpad region of interest (ROI) was quantified weekly. D. The total flux measured from infected footpads was plotted over 9 weeks of infection. Data shown represent the mean flux and SD from groups of four mice. The dotted line indicates the average background flux emitted from uninfected footpads measured 1 week post infection (*n* = 12). A significant difference in the mean total flux emitted between the footpads of mice infected with untreated and rapamycin‐induced parasites was observed at 5 and 9 weeks post‐infection (2‐way ANOVA, ***p ≤ 0.001; **p ≤ 0.005). E. PCR amplification of the floxed *CRK3* locus of Δ*crk3*::*DICRE*/Δ*crk3*::*CRK3* ^Flox^ [*SSU RE9H*] + Rap after purification of amastigotes from the footpads of 10‐week infected mice. Cells were propagated *in vitro* to obtain sufficient genomic DNA for PCR analysis. F. Footpad sizes were recorded by weekly caliper measurement. Data shown represent the mean footpad size and SD from groups of four mice (Unpaired *t*‐test *p ≤ 0.05).

The lack of a conditional system to regulate expression of essential genes is a major obstacle for *in vivo* studies of essentiality, with such studies having crucial applications for drug target validation. To address this we tested if CRK3 activity is essential for survival of the parasite over the course of *in vivo* infection. Monitoring infection by detection of the light signal emitted from bioluminescent *Leishmania* using an *in vivo* imaging system (IVIS) is an established, longitudinal and noninvasive method to correlate signal with pathogen load (Lang *et al*., 2005; Lecoeur *et al*., 2007; Talmi‐Frank *et al*., 2012; Vasquez *et al*., 2015). To assess the outcome of CRK3 loss on the proliferation of *L. mexicana in vivo*, bioluminescent lines were generated by transfection of *L. mexicana* wild‐type and Δ*crk3*::*DICRE*/Δ*crk3*::*CRK3* ^Flox^ promastigotes with a ribosomal integration construct encoding red‐shifted firefly luciferase, Ppy RE9H (Branchini *et al*., 2010; McLatchie *et al*., 2013). Both lines were bioluminescent as determined by luciferase expression assays on logarithmic stage promastigotes. The resulting Δ*crk3*::*DICRE*/Δ*crk3*::*CRK3* ^Flox^ [*SSU RE9H*] cell line produced five fold higher bioluminescence compared with the wild‐type [*SSU RE9H*] control (Fig. S4). Footpad bioluminescence detected with an *in vivo* imaging system (IVIS) correlated well with parasite burden in mice infected with *L. mexicana* expressing Ppy RE9H (Fig. 5A; *y* = 4.8 + 0.43*x*, *R* ^2^ = 0.743 and *p* < 0.0001). The slope of the linear regression line (0.43) revealed smaller increases in bioluminescence with increasing parasite burden. This may be related to tissue absorbance of light *in vivo* or limited substrate availability with increasing numbers of amastigotes within the lesion. Nevertheless, these data show that parasite burdens can be predicted from bioluminescence and that IVIS could be used for the non‐invasive monitoring of parasite growth in mice over 10 weeks of infection. Following treatment of Δ*crk3*::*DICRE*/Δ*crk3*::*CRK3* ^Flox^ stationary phase promastigotes with rapamycin for 24 h the amplification of a 1.36 kb fragment (Fig. 5B) indicated that the majority of parasites had successfully excised floxed *CRK3*. The presence of small amounts of a 3.4 kb amplicon corresponding to the intact floxed *CRK3* gene, however, also suggested that some parasites had retained the gene. These stationary phase Δ*crk3*::*DICRE*/Δ*crk3*::*CRK3* ^Flox^ [*SSU RE9H*] promastigotes either rapamycin treated (+Rap) or not treated (−Rap) were then inoculated into the footpads of BALB/c mice. The *in vivo* bioluminescence in footpads of mice infected with the rapamycin‐treated Δ*crk3*::*DICRE*/Δ*crk3*::*CRK3* ^Flox^ [*SSU RE9H*] was significantly reduced compared to the uninduced control by 5 weeks post‐infection (*p* < 0.001) and this continued up to 9 weeks post‐infection (*p* < 0.005) (Fig. 5C and D). From 5 to 9 weeks the bioluminescence from footpads infected with rapamycin‐treated parasites increased 100 fold and was likely because of the proliferation of parasites that had not responded to rapamycin treatment and persisted in the lesion. To investigate this possibility, viable amastigotes were purified from the lesions of four mice at 10 weeks post‐infection and analyzed for the presence of *CRK3* ^Flox^ by PCR after a single round of *in vitro* culture (Fig. 5E). A 3.4 kb PCR product containing *CRK3* was amplified from all samples, indicating the persistence of parasites that had escaped diCre mediated excision of *CRK3*.

The ability of *CRK3* deficient promastigotes to establish infection was further assessed by measuring footpad sizes at weekly intervals (Fig. 5F). The footpad sizes of mice infected with either untreated or rapamycin‐treated Δ*crk3*::*DICRE*/Δ*crk3*::*CRK3* ^Flox^ [*SSU RE9H*] parasites were similarly low until about 4 weeks post‐infection. Subsequently, footpads containing untreated parasites increased steadily over the course of infection, whilst those infected with rapamycin‐treated Δ*crk3*::*DICRE*/Δ*crk3*::*CRK3* ^Flox^ [*SSU RE9H*] remained low until 9 weeks post‐infection. Comparison of the bioluminescence and lesion sizes suggest that there is a delay in lesion development despite parasite proliferation and that the lesions only increase significantly when parasite load reaches a certain level (equating to bioluminescence ≈10^7^ photons/s); in the case of the untreated parasites this occurred from about 5 weeks while for rapamycin‐treated parasites this level of parasite burden had still not been reached by 9 weeks. Altogether these data show that loss of active CRK3 impairs the establishment of infection *in vivo*, and that a later resurgence of parasites likely results from a small population of cells which previously escaped *CRK3* conditional deletion.

## Discussion

We have developed an inducible system for the genetic manipulation of essential genes in *Leishmania*. Inducible diCre was used to demonstrate the requirement for CRK3 activity in the regulation of mitosis. A distinct growth defect was observed 48 h after induced deletion of *CRK3* (Fig. 2) resulting in cells arrested in G2/M, as well as an accumulation of zoids and eventually a population of enlarged, multi‐flagellated cells (Fig. 3). This phenotype was rescued by expression of a *CRK3* transgene from the ribosomal locus, confirming that loss of CRK3 caused mitotic arrest (Fig. 4). Arrest in G2/M and the accumulation of zoids have previously been reported following incubation of *L. mexicana* promastigotes with the CRK3 inhibitors flavopiridol (Hassan *et al*., 2001) and indirubin (Grant *et al*., 2004), showing correlation between genetic and chemical downregulation of CRK3 activity. In *Trypanosoma brucei* RNAi knockdown of the syntenic orthologue of *CRK3* in the procyclic form also results in G2/M arrest and zoid formation (Tu and Wang, 2004), with the accumulation of such aberrant cells explained by the lack of a checkpoint controlling exit from mitosis and entry in cytokinesis (Hammarton *et al*., 2003; Ploubidou *et al*., 1999). Inducible deletion of *CRK3* indicates that this checkpoint is also absent in *L. mexicana* promastigotes, resulting in impairment of mitotic progression, followed by re‐initiation of G1 in the absence of cytokinesis. It appears that these abnormal cells can eventually undergo cytokinesis; however the daughter cell lacks a nucleus and is often multi‐flagellated (see bi‐flagellated zoid in Fig. 3A), whilst the high levels of cell death occurring 72 h after gene loss show that such progeny are not viable.

CRK3 is active at different stages in the cell cycle by forming complexes with cyclin partners such as CYC6 and CYCA, therefore CRK3 deletion could impact the cell cycle at multiple stages. RNAi of the CYC6 in *T. brucei* procyclic forms results in growth arrest within 48 h of induction and the accumulation of zoids and cells in G2/M (Hammarton *et al*., 2003). A similar phenotype was found in this study with *CRK3* inducible deletion, suggesting that the CRK3:CYC6 complex is involved in regulation of mitosis (Walker *et al*., 2011). Less is known about the activity of CRK3:CYCA. Protein expression assays of *L. donovani* CYC1 (the functional orthologue of CYCA) demonstrates an increased abundance during S‐phase (Banerjee *et al*., 2006) coupled with histone phosphorylation by an active CRK3:CYC1 complex (Maity *et al*., 2011), which is suggestive of S‐phase kinase activity. Active, recombinant *L. mexicana* CRK3:CYCA has also been engineered, with phosphorylation of the T‐loop residue T178 by the CDK activating kinase (CAK) Civ‐1 increasing activity (Gomes *et al*., 2010). The T178 residue is essential for CRK3 activity as T178E mutagenesis inhibits functional rescue in *S. pombe* (Wang, 1998) and ablates kinase activity in recombinant CRK3^T178E^:CYCA (Gomes *et al*., 2010). The necessity of T178 was tested directly in this study, with excision of floxed *CRK3* in the Δ*crk3*::*DICRE*/Δ*crk3*::*CRK3* ^Flox^ [*SSU CRK3* ^T178E^] line leading to cell cycle arrest in G2/M and zoid formation. The growth rate of this line and Δ*crk3*::*DICRE*/Δ*crk3*::*CRK3* ^Flox^ [*SSU CRK3*] were reduced when compared to Δ*crk3*::*DICRE*/Δ*crk3*::*CRK3* ^Flox^ (Table 1), indicative of generally reduced growth rate when expressing a transgene. Episomal complementation with *CRK3* did not result in an observable growth defect (Hassan *et al*., 2001), but this may result from the modulation of the number of episomal copies, as has been observed previously following complementation of the essential *MCA* gene (Ambit *et al*., 2008). Integration into the 18s rRNA locus results in consistently high levels of expression (Misslitz *et al*., 2000) leading to nonphysiological levels of CRK3 and subsequent CRK3:CYC6 activity at potentially inappropriate stages of the life cycle.

The reduced growth rate of promastigotes overexpressing CRK3^T178E^ is likely because of a partial dominant negative effect, whereby inactive CRK3^T178E^ binds endogenous CYC6 leading to impaired protein kinase activity even in the presence of active CRK3. This reduced growth rate may explain both the cell cycle arrest at 72 h in the [*SSU CRK3* ^T178E^] complemented line (Fig. 4A) compared to arrest at 48 h in Δ*crk3*::*DICRE*/Δ*crk3*::*CRK3* ^Flox^ (Fig. 2B) and additionally the lower proportion of zoids when analyzed by flow cytometry (Fig. 4C). The accumulation in G2/M suggests that mutation ablates CRK3:CYC6 activity, rather than CRK3:CYCA, where an increase of cells in G1/S might be anticipated. Both induced and uninduced Δ*crk3*::*DICRE*/Δ*crk3*::*CRK3* ^Flox^ [*SSU CRK3* ^T178E^] have dramatically reduced flagellum length and are immotile (Fig. 4D). The reduced size of the flagellum and a growth defect are similar phenotypes to those observed in cell lines deficient in *ATG5*, a key component of the autophagic pathway (Williams *et al*., 2012). This is likely a result of their impaired ability to salvage material through the autophagic pathway, imparting selection on the parasites to reduce energy through flagellum regression. The partial dominant negative effect of CRK3^T178E^ may also result in metabolic stress in these cells leading to the phenotype observed. The importance of T178 as an active site residue for regulating progression through G2/M implicates upstream modifiers of this residue as essential regulators of the *L. mexicana* cell cycle. In mammalian cells CDK7 acts as a CAK to regulate CDK1 by phosphorylation at this T‐loop residue, yet no CAK homologues have been identified in the *Leishmania* genome (Gomes *et al*., 2010). The identification of potential post‐transcriptional modifiers of the CRK3 T‐loop residue that act in an analogous fashion to CDK7 would therefore yield promising targets for drug discovery. The phenotype of the induced cell line shows the importance of the T‐loop residue for CRK3 activity and mitotic function within the cell, endorsing this complementation assay as a rational approach for active site investigation.

The assessment of gene essentiality for amastigote viability is an important approach in the context of drug target validation as this life cycle stage is the pathologically significant form. The recent utilization of plasmid shuffle has facilitated the study of *Leishmania* genes involved in life cycle differentiation and essentiality both in amastigote and promastigote forms by the generation of partial *null* mutants (Dacher *et al*., 2014; Morales *et al*., 2010). Retention of an episomal gene in a *null* mutant cell line after murine infection is a useful approach to assess that gene as necessary to amastigote survival *in vivo* (Wiese, 1998). Despite such elegant utilization of reverse genetic methods to probe gene function, no method exists for the generation of conditional *null* mutants during *in vivo* infection. Our study does not address this lack directly because of the sensitivity of amastigotes to rapamycin, however as diCre activity remains high in stationary‐phase promastigotes *CRK3* was efficiently excised (Fig. 5B) to probe the subsequent infectivity of CRK3‐deficient promastigotes. By tracking the progression of infection with reporter parasites expressing the highly sensitive red‐shifted luciferase (Branchini *et al*., 2010; McLatchie *et al*., 2013) and by footpad size measurement, we demonstrate that the *CRK3*‐deficient *L. mexicana* are unable to proliferate in their mammalian host (Figs. 5C, D, and F). Importantly, the wild‐type line expressing luciferase grows normally in mice following rapamycin treatment, which indicates that lack of growth of the *CRK3*‐deficient mutant is not a result of the drug treatment. The average light intensities emitted from footpads infected with the wild‐type [*SSU RE9H*) line and those from footpads infected with the Δ*crk3*::*DICRE*/Δ*crk3*::*CRK3* ^Flox^ [*SSU RE9H*] line retaining floxed *CRK3* are at similar levels throughout infection, yet mean footpad size is larger in wild‐type [*SSU RE9H*] infected mice after 3 weeks post‐infection; such disagreement may be a result of the five fold lower signal intensity of the wild‐type [*SSU RE9H*] compared with the experimental line (Fig. S5) and therefore an overall higher burden of the wild‐type line is likely masked by a reduced bioluminescent signal intensity.

Interestingly, parasite burden as measured by total flux remains consistently above the background intensity (dashed line, Fig. 5D) in those footpads infected with the *CRK3*‐deficient line, suggestive of the survival of a low number of bioluminescent parasites. The outgrowth of these parasites was observed through an increased bioluminescence signal at 9 weeks post‐infection compared with 5 weeks (Fig. 5C and D). Purification and PCR analysis of these parasites show they retained the floxed *CRK3* (Fig. 5E) and that the persistence of signal and subsequent increase are a result of incomplete excision of floxed *CRK3* during the 24 h incubation with rapamycin. These data further demonstrate the essentiality of *CRK3* activity for establishing infection.

This is the first time an essential gene in promastigotes has been studied *in vivo* by conditional deletion, representing a useful tool to probe gene function. We are validating the feasibility of conditional gene deletion *ex vivo* and *in vivo* using rapamycin and nonimmuno‐inhibitory rapamycin analogues (‘rapalogs’), with such work being useful for the future of drug target validation. DiCre activity has been demonstrated *in vivo* (Jullien *et al*., 2007), however rapamycin treatment may be a limitation because of influence on the host immune response and on amastigote proliferation. Our attempts to study the effect of *CRK3* ^Flox^ deletion in lesion‐derived amastigotes grown in axenic culture medium was problematic because of reduced proliferation of both experimental and wild‐type *L. mexicana* at the relatively low dose of 50 nM rapamycin, therefore the use of rapalogs would be a rational approach for induction of diCre activity if they have reduced binding affinity for *Leishmania* TORs (Madeira da Silva and Beverley, 2010). A second generation diCre is currently in development and may present an alternative method for inducible gene deletion *in vivo*. In diCre2, each subunit is fused to mutant FKBP domains that are dimerised by the rapalog AP20187, which is amenable to *in vivo* use (Collins *et al*., 2013). Such a system could be applied for use in *Leishmania* and would complement our existing floxed gene replacement approach.

In conclusion we have developed a highly efficient inducible gene deletion system that when used with transgene complementation allows for the first time the function of essential *Leishmania* genes to be elucidated. We have applied this approach to show that CRK3 is required for promastigote progression through mitosis, with gene deletion mutants showing a G2/M arrest and an accumulation of zoids, indicative of a lack of a cell cycle checkpoint in cytokinesis. Inducible deletion of CRK3 in stationary phase promastigotes attenuates infection in a murine host, providing further genetic validation of CRK3 as a potential drug target (Gomes *et al*., 2010; Grant *et al*., 1998; 2004; Hassan *et al*., 2001; Walker *et al*., 2011). Our diCre method provides a powerful tool for analysing genes essential for promastigote proliferation and to the study of the differentiation of promastigotes to amastigotes.

## Experimental procedures

### Ethics statement

Animal studies were carried out under UK Home Office regulations (Project licence PPL 60/4442).

### Parasite culture and transfection

*Leishmania mexicana mexicana* (MNYC/BZ/62/M379) promastigotes were cultured at 25°C in HOMEM supplemented with 10% heat inactivated foetal calf serum (HI‐FCS) and 1% penicillin/streptomycin (PEN/STREP). Amastigotes were cultured in Schneider's Insect Medium supplemented with 20% HI‐FCS, 1% PEN/STREP and 15 μg mL^−1^ Hemin at pH5.5. Mid‐log phase *L. mexicana* promastigotes were transfected with 10 μg of digested DNA by electroporation using the Nucleofector system with the Human T‐Cell kit (Lonza) as described previously (Castanys‐Muñoz *et al*., 2012). Transgenic cell lines were grown in the presence of appropriate antibiotics at the following concentrations: G418 50 μg mL^−1^, blasticidin 10 μg mL^−1^ and puromycin 10 μg mL^−1^ (InvivoGen).

### Construct design and development

A full list and descriptions of all primers (Table S1) and plasmids (Table S2) used in this study are available. To produce a diCre expression vector, the diCre coding sequences Cre59‐FKBP12 and Cre60‐FRB were each flanked by actin and β‐tubulin sequences in array with blasticidin resistance cassette flanked by *DHFR‐TS* regulatory elements. The sequence was synthesized and sub‐cloned into the pDONR221 vector (GenScript). The backbone of the loxP vector containing the loxP sites flanking a multiple cloning site and other restriction enzyme regions flanked by regulatory elements was synthesized (GenScript). The *PAC*, *mCherry* and *CRK3‐GFP* cassettes were inserted by enzymatic restriction digest mediated ligation, and subsequently sub‐cloned into pDONR221. Addition of *CRK3* homology flanking homology was performed by MultiSite Gateway 3‐fragment vector construction (Invitrogen) as per manufacturers’ guidelines. Briefly, flanks were amplified by PCR by Phusion polymerase (New England BioLabs) using oligonucleotides conferring *attB* recombination sites to the amplicons. Subsequent BP reactions inserted the flanks into appropriate pDONR vectors containing *attL* sites for site‐specific recombination. An LR reaction resulted in the flanking of diCre and loxP vectors into a pDEST vector for transfection. Finally, complementation plasmids were generated by inserting the *CRK3*, *CRK3* ^T178E^ and *RE9H* genes (Branchini *et al*., 2010; McLatchie *et al*., 2013) into a modified version of pGL631 (Misslitz *et al*., 2000) containing a G418r cassette for SSU integration construct by *Xho*I & *Not*I restriction enzyme digestion and ligation.

### Induction of diCre mediated gene deletion

All experiments were conducted using cells in the early to mid log stage of exponential growth (between 1 and 5 × 10^6^ cells mL^−1^) with the exception of the stationary phase inducible gene deletion. Between 1 nM and 1 μM rapamycin (Abcam) was administered by inoculation into the cell culture medium from a 100 μM working stock.

### Conditional gene deletion analysis

Taq polymerase (NEB) was used to PCR amplify the regions surrounding *GFP* ^Flox^ and *CRK3* ^Flox^ using primers shown in Table S1 and a *T*_A_ calculated using an online *T*_m_ calculator (New England BioLabs) and 30 cycles for amplification.

### Western blot analysis

For western blotting analysis, either 1 × 10^7^ cells were loaded per lane or equal concentrations of protein extract as quantified by Bradford assay of a 10% NuPAGE Bis‐Tris gel (Invitrogen) in MOPS running buffer and transferred onto Hybond‐C nitrocellulose membranes (GE Healthcare). Primary antibodies against GFP were used to detect GFP and CRK3‐GFP expression at 1:1000 whilst anti‐EF1α was used as a loading control at 1:5000. Membranes were washed three times in TBST, incubating for 10 min each time, before incubation with horse radish peroxidase (HRP)‐conjugated secondary rabbit and mouse antibodies at 1:5000 dilution for 1 h at room temperature. After washing three times in TBST, the membrane was treated with an ECL (enhanced chemiluminescence) kit (SuperSignal West Pico Chemoluminescent Substrate, Pierce) according to manufacturer's instructions and then exposed on Kodak photographic film.

### Infection of mice

BALB/c mice were purchased from Charles River (MA., USA) and infected in the right footpad with 2 × 10^6^ stationary‐phase *L. mexicana* promastigotes in 1 × PBS. Lesion size was monitored weekly and Δ*crk3*::*DICRE*/*CRK3* [*SSU GFP* ^Flox^] amastigotes were purified before the lesions reached a thickness of 5 mm.

### Purification of lesion‐derived amastigotes

Lesion‐derived Δ*crk3::DICRE/CRK3* [*SSU GFP* ^Flox^] amastigotes were purified by homogenizing the extracted lesion in 1xPBS and passing the solution through a 20 μm cell strainer. Amastigotes were pelleted by centrifugation at 2000 g for 10 mins, followed by re‐suspension in culture medium. To prevent cells from clumping together and ensure accurate cell counting, amastigote cultures were first centrifuged at 2000 g for 10 mins and the supernatant removed to leave the pellet in 500 μL volume. The pellet was re‐suspended in this volume by gentle syringing through a blunt 16G needle and the single cell suspension added back to the culture medium. Cell counting was performed by mixing the homogenized culture 1:1 with Trypan blue and cell counting with a Haemocytometer (Neubauer).

### Macrophage differentiation and amastigote infection

Nondifferentiated monocytes were extracted from the femurs and tibia of BALB/c mice by dissection to remove the bones. RPMI 1640 medium was used to wash the bone marrow out of the intact bones by syringing with a 25G needle. Extracted cells were quantified by dilution in Trypan blue (1:1) and counting with a haemocytometer. Monocytes were seeded at 5 × 10^5^ cells mL^−1^ in MΦ Medium (DMEM + L‐Glut + 20%FCS + 1% P/S + 30% L‐Cell M) in 8 mL volumes in Petri dishes and incubated at 37°C with 5% CO_2_ for 3 days to induce differentiation to monocyte‐derived macrophage. After this period the medium was replaced and by day 5 the cells were removed from the dishes using a cell scraper with ice‐ cold RPMI 1640. Bone‐marrow derived macrophages were adhered at a concentration of 5 × 10^5^ cells mL^−1^ overnight in DMEM medium with 10% HIFCS at 37°C in 5% CO_2_ onto 8‐chamber tissue culture slides (LAB‐TEK) for microscopic analysis or 12 well plates for DNA extraction and flow cytometry analysis. Macrophages were then infected at a ratio of five parasites per macrophage with lesion‐derived Δ*crk3::DICRE/CRK3* [*SSU GFP* ^Flox^] amastigotes, which had been previously grown in axenic medium in the presence or absence of rapamycin for 24 h. Wells were washed at 24 h post‐infection to remove extracellular parasites and media replenished with DMEM/10% HIFCS. Cells were removed from the plates for DNA extraction and flow cytometry analysis by gentle scraping in ice cold RPMI at the 120 h end time point.

### Fluorescence microscopy analysis

For imaging, 2 × 10^6^ parasites were washed in 1 × PBS, re‐suspended in Fluoromount‐G (SouthernBiotech) DAPI infused mounting medium and mounted on glass slides for analysis. Parasite morphology was observed by DIC and mCherry fluorescent imaging, and DNA content observed by DAPI fluorescent imaging using a Delta Vision core (Image Solutions) inverted microscope equipped with mCherry and DAPI filter sets. Images were processed using Photoshop CS (Adobe) image software. GFP expression of intracellular amastigotes was assessed by fluorescent microscopy. Cells were imaged between 24 and 120 h after infection in the DeltaVision Core environmental chamber at 37°C and 5% CO_2_ upon incubation in 1 × PBS infused with DAPI.

### DNA content and GFP expression analysis by flow cytometry

Parasites were prepared for DNA content analysis as described previously (Paul Hassan *et al*., 2001) with the exceptions that a MacsQuant flow cytometer was used to analyze 100,000 cells per sample. Cell distribution was modelled using FlowJo software (Tree Star). For determining GFP expression of promastigotes and amastigotes by flow cytometry analysis, live cells were washed twice in 1xPBS and passed through a nitex mesh prior to acquisition.

### Viability assay

Log‐phase promastigotes were seeded at 5 × 10^5^ cells mL^−1^ and grown in the presence or absence of 100 nM rapamycin. At 72 h post‐treatment 1 × 10^7^ cells were washed once with 1 × PBS and incubated with 5 ug mL^−1^ propidium iodide (PI) for 15 minutes at room temperature in the dark. A heat lysed (HL) control in which half the sample was lysed by incubation at 70°C for 3 min was included to enable an appropriate live/dead gate to be drawn. Cells were washed with 1 × PBS and used to acquire 100,000 events per group by flow cytometry using a MacsQuant flow cytometer.

### *In vivo* imaging

For imaging, mice were anaesthetized with 4.0% isofluorane/1.5 L O_2_ per minute and inoculated by subcutaneous injection with 200 μL of D‐luciferin (15 mg mL^−1^ in Mg/Ca‐free Dulbecco's modified PBS). Light emission was recorded 10 min after inoculation using an IVIS Spectrum bioluminescence imaging system (PerkinElmer). Imaging was performed with an open emission filter, for 30–60 s exposures, large binning, and 1 f/stop, and captured with a charge‐coupled device (CCD) camera. The absolute unit of photon emission was given as radiance (photons/second/cm^2^/steradian). Images were analysed using Living Image Software (PerkinElmer) and regions of interest (ROI) of equal size were selected over the infected footpads to quantify the amount of photon emission as total photon flux in photons per second (photons/s).

### Statistical analysis

Statistical analysis was performed using GraphPad Prism 5. The analysis of significance of the data was performed by 2‐way ANOVA when comparing data from induced (+Rap) and uninduced (−Rap) Δ*crk3*::*DICRE*/Δ*crk3*::*CRK3* ^Flox^ [*SSU RE9H*] infections and by paired *t*‐test when comparing footpad sizes.

## Supporting information

Supporting Information
