# Supplementary material for: Conditional gene deletion with DiCre demonstrates an essential role for CRK3 in L eishmania mexicana cell cycle regulation
Source: Mol Microbiol. 2016 Apr 13;100(6):931–44. doi: 10.1111/mmi.13375 (PMC4913733; doi:10.1111/mmi.13375)
Supplement: Supplementary file 1 — Supporting Information [file MMI-100-931-s001.pdf]

Figure S1

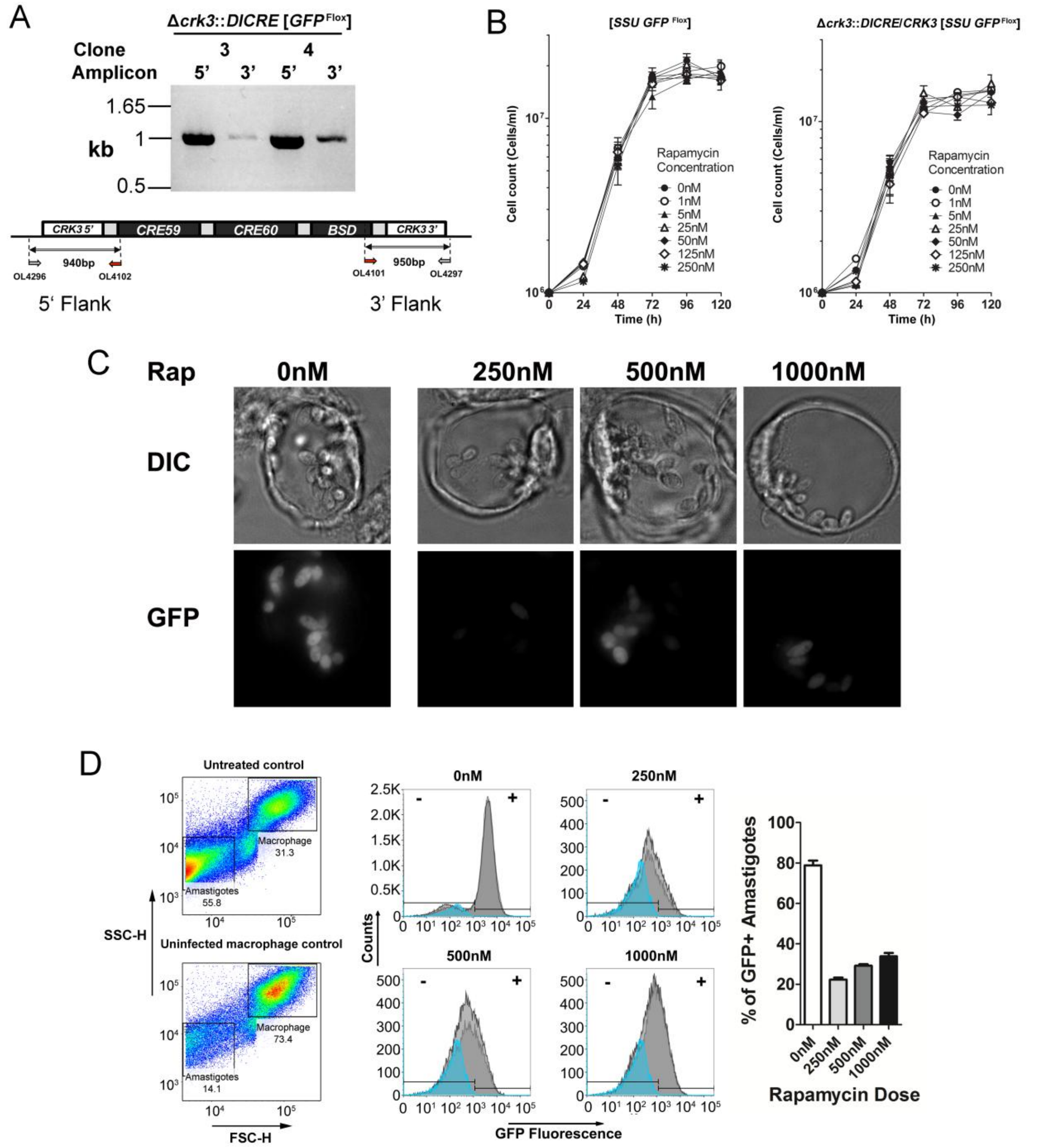

Fig. S1. A. Replacement of a single copy of *CRK3* by diCre construct integration into the [*SSU GFP* ^{Flox}] cell line was confirmed by PCR amplification of genomic DNA extracted from two clones (3 and 4). Oligonucleotides (OL) that bind outside the integration site (grey arrows) and within the diCre coding sequence (red arrows) were used to amplify 940 bp and 950 bp amplicons. Clone 3 was designated as the experimental line Δ*crk3::DICRE/CRK3* [*SSU GFP*^{Flox}].

B. Experimental Δ*crk3::DICRE/CRK3* [*SSU GFP*^{Flox}] or control [*SSU GFP*^{Flox}] *L. mexicana* promastigotes were seeded at $1x10^6$ cells ml^{-1} and incubated in the presence or absence of between 1 to 250 nM rapamycin. Cell density was determined at 24 hour intervals by cell counting (N=1-3 technical replicates, error SEM).

C. Representative DIC (upper) and GFP (lower) images from live cell imaging of amastigotes-infected macrophages at 5 days post-infection. GFP expression from live amastigotes was imaged using a Delta Vision core fluorescent microscope.

D. GFP intensity loss in amastigotes extracted at day 5 post *in vitro* macrophage infection; (left) amastigotes were gated from large, granular macrophage by forward scatter (FSC) for size and side-scatter (SSC) for granularity. (middle) Histograms of amastigote GFP intensity were generated from amastigote gates with retention of GFP expression at $>10^3$ fluorescence intensity based on rapamycin untreated controls. Blue plots represent the amastigote gate plotted from a macrophage only control group to represent background cellular ‘debris’ as a result of macrophage lysis following sample preparation (left). >20,000 amastigote events were analysed per treatment group based on two biological replicates shown as dark and light grey plots. (right) Retention of GFP signal as a % of amastigote gate displayed as bar graphs for each treatment group (Data represent means ± SEM).

Figure S2

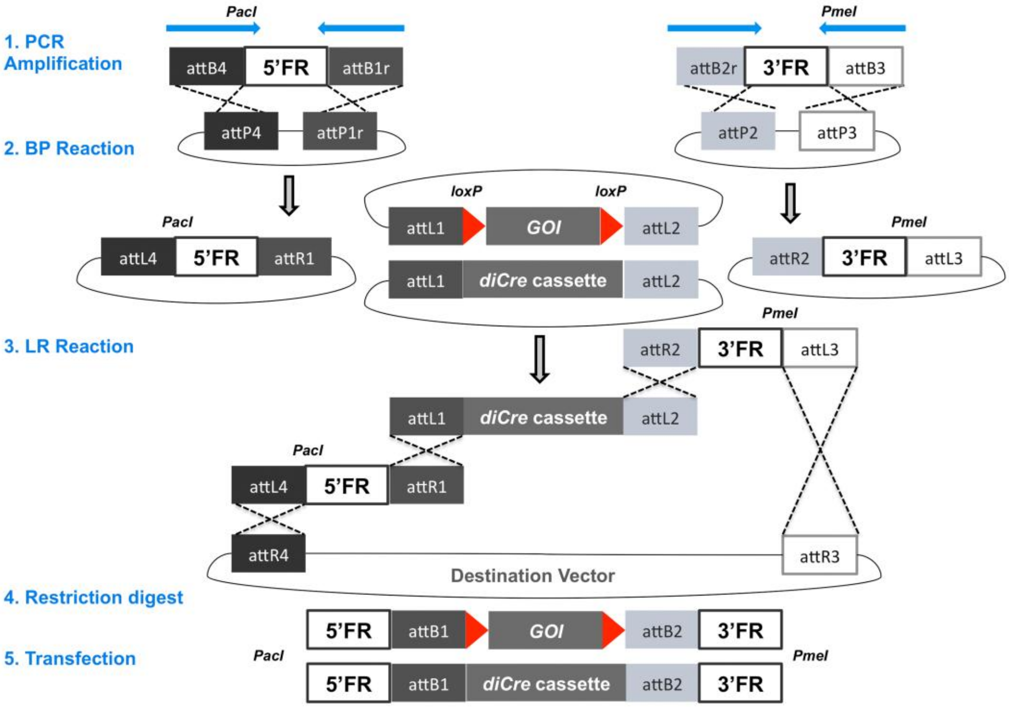

Fig. S2. Pipeline of Gateway-mediated addition of target gene homologous flanks to diCre and loxP vectors. (*1*) Primers (blue arrows) containing appropriate att sites and 5' *Pac*I or 3'*Pme*I unique restriction sites amplify a 0.5-1 kb region up- and downstream of the gene. (*2*) BP clonase catalyses the insertion of these flanks into their appropriate vectors. (*3*) The resulting 5', 3' and diCre or loxP vectors are recombined into a pDEST vector by LR clonase. (*4*) The final vector is linearised by *Pac*I and *Pme*I digest for (*5*) transfection into *L. mexicana*. This method enables flanking of both the floxed gene of interest (GOI) expression cassette and diCre expression cassette.

Figure S3

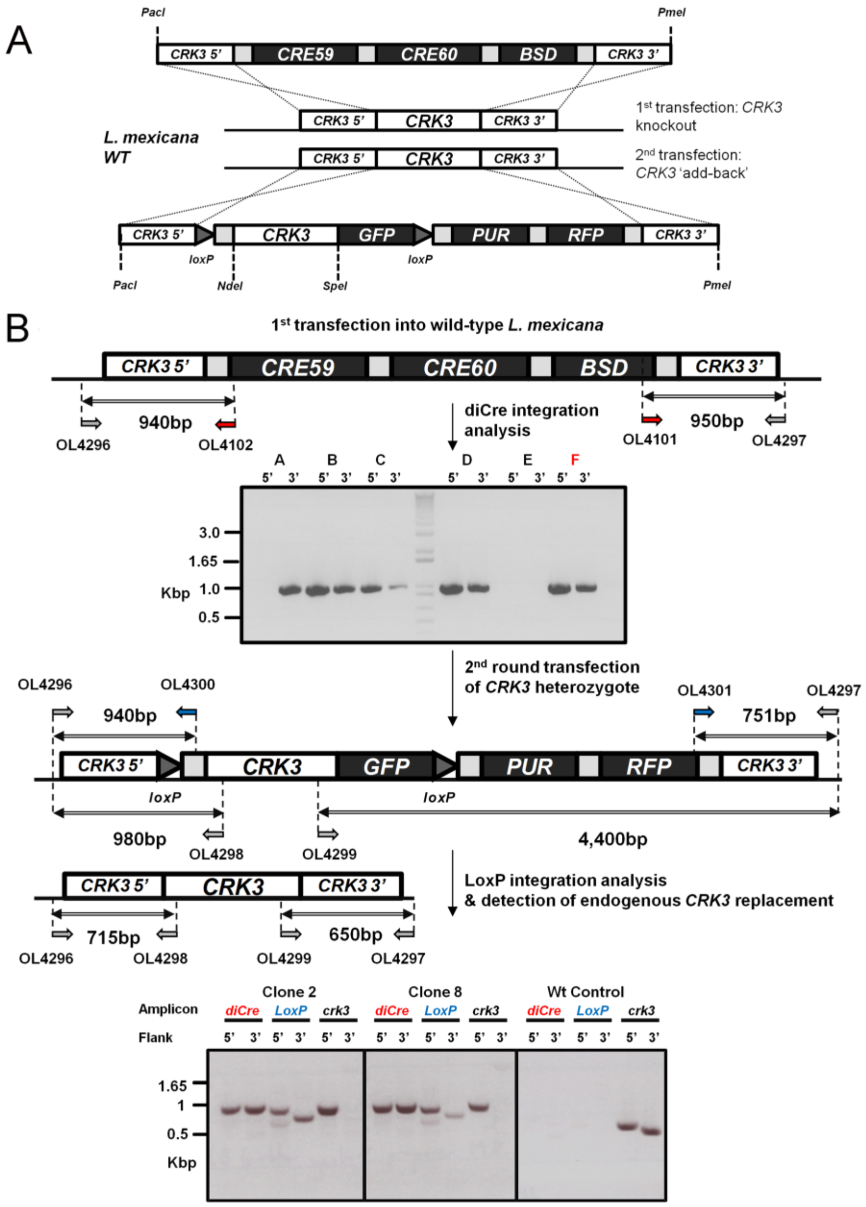

Fig. S3. A. Schematic representing the diCre and floxed CRK3 replacement strategy. Homologous recombination was facilitated by Gateway flanking of both diCre and loxP vectors with ~500 bp of *crk3* 5' and 3' homologous regions to replace both alleles.

B. Transfection of wild-type *L. mexicana* with the diCre construct: integration was confirmed by PCR amplification of genomic DNA extracted from six clones with oligonucleotides (OL) binding outside the integration site (grey arrows) and within the diCre coding sequence (blue arrows) to amplify 940 and 950 bp amplicons. A single blasticidin (BSD) resistant clone F with *diCre* integrated at the *crk3* locus was subsequently transfected with the loxP construct to replace the remaining endogenous *crk3* allele with a floxed *CRK3* fused to a 3' *GFP* tag, thereby generating a diCre-mediated conditional deletion line: Δ*crk3*::*DICRE*/

Δ*crk3*::*CRK3*^{Flox}. PCR amplification of genomic DNA extracted from two blasticidin/ puromycin (PUR) double resistant clones (2 and 8) with oligonucleotides binding outside the integration site (grey arrows), within the *crk3* coding sequence (grey arrows), within the loxP vector (blue arrows) and diCre sequences (red arrows).

Figure S4

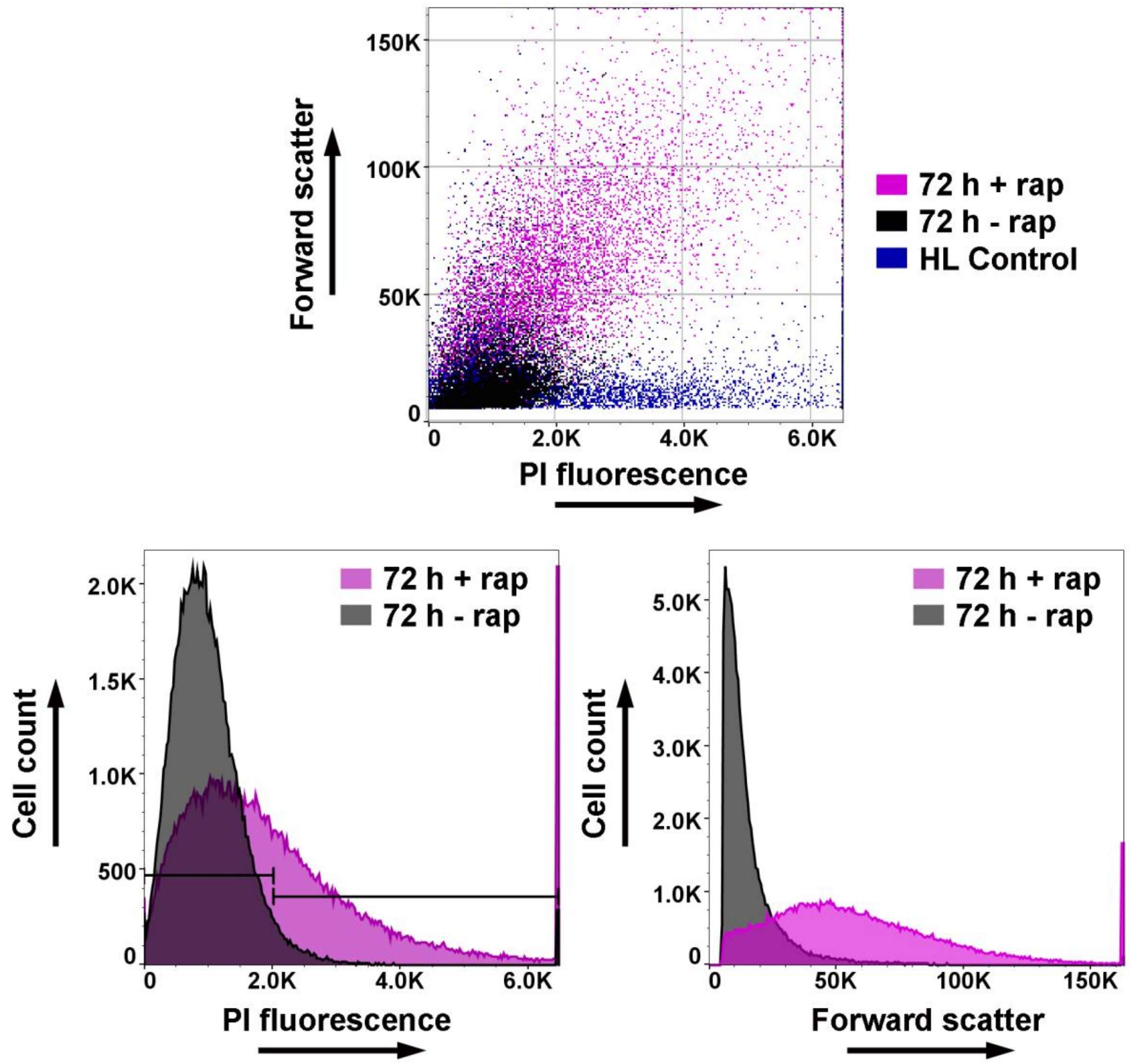

Figure S4. Viability assay of Δ*crk3*::*DICRE*/Δ*crk3*::*CRK3*^{Flox} promastigotes. Cells were grown in the presence or absence of 100 nM rapamycin for 72 h. Live cells were incubated with 5ug ml^{-1} propidium iodide (PI) for 15 minutes and uptake measured by flow cytometry alongside a heat lysed (HL) control in which half the cells were lysed by incubation at 70°C for 3 min prior to flow cytometry analysis. Top panel shows cell size as measured by forward scatter in the y-axis and cell lysis by increasing PI fluorescence along the x-axis. Bottom left panel shows the gating strategy whereby cells are defined as + or – in PI uptake based on the HL control. Bottom right panel is an analysis of promastigote cell size following incubation in the presence or absence of rapamycin. Results are representative of 2 independent experiments.

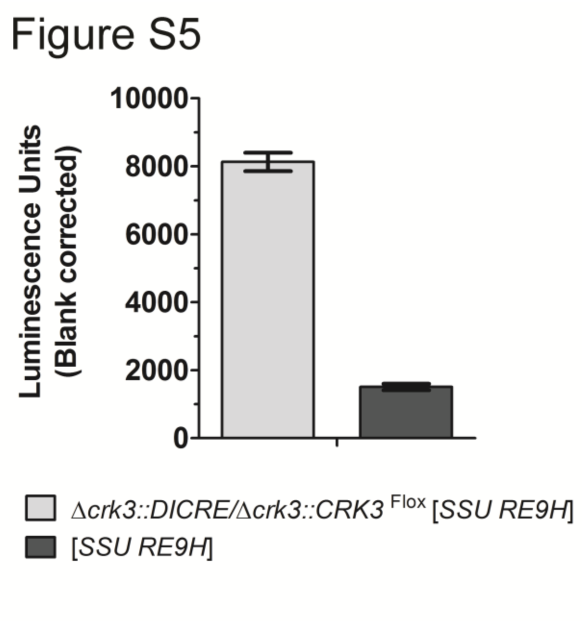

Figure S5. *In vitro* bioluminescence expression assay of experimental and control promastigotes. Promastigotes were assayed during logarithmic growth and luminescence expression data was acquired 30 minutes post luciferin treatment. Error bars represent the SEM of two technical replicates per clone.

Table S1

Oligo No.		Description	Sequence
**Gateway cloning of CRK3 homologous flanks**			
OL4249	F	Amplification of a 5’ *CRK3* homologous flank with attB4/P1r	GGGGACAACTTTGTATAGAAAAGTTGCCCTTAATTAAAAAGGTAGAGGATGCCGTTTT
OL4250	R		GGGGACTGCTTTTTTGTACAAACTTGCTTGAAATGTTGCAGGGAGAAA
OL4251	F	Amplification of a 3’ *CRK3* homologous flank with attB2r/B3	GGGGACAGCTTTCTTGTACAAAGTGGGGAGTGGAAAAGGCATGACTGAA
OL4252	R		GGGGACAACTTTGTATAATAAAGTTGCGGTTTAAACTTTCCTCCCCAGCACGCACAC
**Generation of *CRK3* loxP expression and complementation vectors**			
OL4065	F	Amplification of puromycin resistance cassette from pGL631	GATCCTGCAGCGCGTGGATGTCGCGCAG
OL4066	R		GATCGCTAGCCTAGGCACCGGGCTTGCG
OL4293	F	Amplification of SAS-HASPB-*mCherry* from pGL1893 to integrate at reporter site	GATCCTCGAGAATTGCCCGCTTTCCAT
OL4294	R		GATCGCGGCCGCGGGATCCTCAATGATGA
OL4316	F	Amplification of GFP from pGL1773 for integration as N-terminal tag	GATCCATATGATGGTGAGCAAGGGCGAG
OL4317	R		GATCGGTACCCTTGTACAGCTCGTCCAT
OL4318	F	Amplification of 6xHA integration as N-terminal tag	GATCCATATGTACCCTTACGATGTGCCT
OL4319	R		GATCGGTACCTGCGTAATCGGGCACATC
OL4320	F	Amplification of GFP from pGL1773 for integration as C-terminal tag	GATCACTAGTATGGTGAGCAAGGGCGAG
OL4321	R		GATCTCTAGATCACTTGTACAGCTCGTCCAT
OL4541	F	Amplification of SAS-HASPB-mCherry for insertion via *Hind*III: enables the replacement of HASPB-mCherry by *Xho*I and *Not*I	GATCAAGCTTAATTGCCCGCTTTCCATTTCG
OL4542	R		GATCGCGGCCGCGGGATCCTCAATGATGATGAT
OL4067	F	Amplification of the *CRK3* CDS for insertion into the loxP MCS: no Stop codon amplified due to C-terminal *GFP* fusion	GATCCATATGTCTTCGTTTGGCCGTGTG
OL4103	R		GATCATCGATCCAACGAAGGTCGCTGAA
OL4388	F	Amplification of the *CRK3* CDS for insertion into the loxP MCS: Stop codon amplified due to N-terminal *GFP* fusion	GATCACTAGTTCTTCGTTTGGCCGTGTGACC
OL4389	R		GATCTCTAGACTACCAACGAAGGTCGCTGAA
OL4591	F	Amplification of *CRK3-his* for insertion into pGL2277 to generate an 18S RNA integration vector for complementation of the floxed *CRK3* inducible deletion line	CTCGAGATGTCTTCGTTTGGCCGT
OL4592	R		GCGGCCGCCTAATGATGATGATGATGATGCCAACG AAGGTCGCTGAA
OL4601	F	Mutagenesis primers for T178 mutation to a glutamic acid residue to create *CRK3* ^{T178E}	GCACACCTACGAGCACGAGGTGG
OL4602	R		ATGGGCACTTGAAACGCAC
**Primers for analysis of vector integration and floxed gene loss by PCR amplification**			
OL4101	F	Internal forward (BLA) and reverse (FKBP12) primers to detect diCre integration into the genome	CTGGTTATGTGTGGGAGG
OL4102	R		GATGGTTTCCACCTGCAC
OL4287	F	Upstream and downstream primers to amplify the floxed *GFP* fragment to detect gene loss by diCre induction	GCTCGCGTGTGTTGAGCC
OL4288	R		CATTCGTGGGCTCCAGCT
OL4296	F	Primers binding out-with the *CRK3* integration site	GATCGTGGGAAGGGGAAG
OL4297	R		GGAAGTCCAAGTAGCGCG
OL4298	R	Primers binding the *CRK3* gene	GGTCACACGGCCAAACGA
OL4299	F		GCCAAGGAGGCCCTACAG
OL4300	R	Primers binding the loxP vector at the 5’ splice acceptor site (SAS) and 3’ poly-adenylation site (PAS)	GGTGGACGGCTCAACACA
OL4301	F		GTGTGCTGTGCGTTCAGC
OL4781	F	Upstream and downstream primers for amplification of a floxed *CRK3-GFP* fragment to detect gene loss	AACTGGCAGCAGCGATTTGGCAGGGG
OL4782	R		GCACCGTGGGCTTGTACTCGGTCATG
OL4748	F	Primers to check for integration of *RE9H* construct (pGL2398) into the ribosomal locus	TCGTGAGACGCCCAGCGAATG
OL4750	R		ACCGACGCCCACATCGAGGTG

Table S1. A list of the oligonucleotides used in this study.

Table S2

pGL No.	Gene ID	Gene Name	Backbone	Description
2313	N/A	*diCre*	pDONR221	DiCre expression cassette entry vector
2314	N/A	*loxP- C-6xHA*	pDONR221	LoxP (empty) expression cassette: c-terminal 6xHA tag
2315	N/A	*loxP-C-GFP*	pDONR221	LoxP (empty) expression cassette: c-terminal GFP tag
2316	N/A	*loxP-N-GFP*	pDONR221	LoxP (empty) expression cassette: n-terminal GFP tag
2375	LmxM.36.0550	*CRK3*	pGL631	WT *CRK3* ribosomal SSU integration vector
2376	LmxM.36.0550	*CRK3* ^{T178E}	pGL631	Mutated *CRK3*^{T178E} ribosomal SSU integration vector
2398	N/A	*RE9H*	pGL631	Red-shifted luciferase bioluminescent protein in G418r pRib
2445	LmxM.36.0550	*5' CRK3 flank*	pDONR P41-Pr	5' Flank (500bp) ready for Gateway recombination
2446	LmxM.36.0550	*3' CRK3 flank*	pDONR P2r-P3	3' Flank (500bp) ready for Gateway recombination
2455	N/A	*diCre*	pDEST R4-R3	DiCre cassette flanked with *CRK3* homologous arms
2456	LmxM.36.0550	*CRK3*	pDEST R4-R3	*CRK3-GFP* ^{flox} cassette flanked with *CRK3* homology
2461	N/A	*GFP* ^{flox}	pGL631	Floxed *GFP* in pRib: for functional analysis of diCre

Table S2. A list of the plasmids generated in this study.
